# Complete genome sequence of “*Candidatus* Phytoplasma sacchari” obtained using a filter-based DNA enrichment method and Nanopore sequencing

**DOI:** 10.3389/fmicb.2023.1252709

**Published:** 2023-10-02

**Authors:** Rong-Yue Zhang, Xiao-Yan Wang, Jie Li, Hong-Li Shan, Yin-Hu Li, Ying-Kun Huang, Xia-Hong He

**Affiliations:** ^1^Yunnan Key Laboratory of Sugarcane Genetic Improvement, Sugarcane Research Institute, Yunnan Academy of Agricultural Sciences, Kaiyuan, China; ^2^State Key Laboratory for Conservation and Utilization of Bio-Resources in Yunnan, Yunnan Agricultural University, Kunming, China; ^3^School of Landscape and Horticulture, Southwest Forestry University, Kunming, China

**Keywords:** phytoplasma, sugarcane white leaf, sugarcane grassy shoot, genome sequencing, DNA enrichment

## Abstract

Phytoplasmas are phloem-limited plant pathogens, such as sugarcane white leaf (SCWL) phytoplasma, which are responsible for heavy economic losses to the sugarcane industry. Characterization of phytoplasmas has been limited because they cannot be cultured *in vitro*. However, with the advent of genome sequencing, different aspects of phytoplasmas are being investigated. In this study, we developed a DNA enrichment method for sugarcane white leaf (SCWL) phytoplasma, evaluated the effect of DNA enrichment *via* Illumina sequencing technologies, and utilized Illumina and Nanopore sequencing technologies to obtain the complete genome sequence of the “*Candidatus* Phytoplasma sacchari” isolate SCWL1 that is associated with sugarcane white leaf in China. Illumina sequencing analysis elucidated that only 1.21% of the sequencing reads from total leaf DNA were mapped to the SCWL1 genome, whereas 40.97% of the sequencing reads from the enriched DNA were mapped to the SCWL1 genome. The genome of isolate SCWL1 consists of a 538,951 bp and 2976 bp long circular chromosome and plasmid, respectively. We identified 459 protein-encoding genes, 2 complete 5S-23S-16S rRNA gene operons, 27 tRNA genes, and an incomplete potential mobile unit (PMU) in the circular chromosome. Phylogenetic analyses and average nucleotide identity (ANI) and digital DNA–DNA hybridization (dDDH) values based on the sequenced genome revealed that SCWL phytoplasma and sugarcane grassy shoot (SCGS) phytoplasma belonged to the same phytoplasma species. This study provides a genomic DNA enrichment method for phytoplasma sequencing. Moreover, we report the first complete genome of a “*Ca*. Phytoplasma sacchari” isolate, thus contributing to future studies on the evolutionary relationships and pathogenic mechanisms of “*Ca*. Phytoplasma sacchari” isolates.

## Introduction

Phytoplasmas are phloem-limited bacterial plant pathogens that were discovered in 1967 and were initially classified as mycoplasma-like organisms (MLOs) (Doi et al., 1967). MLO being replaced with “phytoplasma” was initially suggested in 1992 at the meeting on the taxonomy of Mollicutes (Tully, 1993). In 2004, different species of phytoplasma were included in the provisional genus “*Candidatus* Phytoplasma” by the IRPCM Phytoplasma/Spiroplasma Working Team-Phytoplasma taxonomy group (2004). However, limited information could be obtained regarding these pathogens because it is difficult to culture them *in vitro*. With the advent of genome sequencing technologies and comparative genome analysis, our understanding of the genetic structure, phylogeny, evolution, metabolic pathways, and possible virulence factors of phytoplasmas has enhanced. The first complete genome sequence of the genus was reported for “*Candidatus* Phytoplasma asteris” OY-M isolate in 2004 (Oshima et al., 2004); 12 complete phytoplasma genomes and 35 draft phytoplasma genomes have been reported so far (Bertaccini et al., 2022; Wei and Zhao, 2022; Kirdat et al., 2023). However, in a previous study, phytoplasma genome sequencing using total DNA generated only 0.17% of Illumina sequencing reads (Cho et al., 2019) because it is difficult to obtain pure phytoplasma genomic DNA due to its unculturable nature, indicating that the enrichment of phytoplasma genomic DNA is essential for sequencing. The main enrichment methods used in previous studies are density gradient centrifugation, pulse field gel electrophoresis (PFGE) (Oshima et al., 2004; Bai et al., 2006; Kube et al., 2008; Chen et al., 2014), methyl-CpG-binding domain-mediated method (Kirdat et al., 2020, 2021; Nijo et al., 2021; Debonneville et al., 2022), and immunoprecipitation-based method (Tan et al., 2021).

Phytoplasmas are naturally transmitted by phloem-feeding insects and cause more than 1,000 types of plant diseases worldwide, resulting in significant economic losses in the agriculture industry (Wang et al., 2022). The varied symptoms of phytoplasma infections include plant dwarfing, leaf yellowing, phyllody, witches' broom, stunting, proliferation, and phloem tissue necrosis (Oshima, 2021; Wang et al., 2022). Phytoplasmas are also responsible for two diseases in sugarcane, namely, sugarcane white leaf (SCWL) and sugarcane grassy shoot (SCGS), which cause heavy losses in several sugarcane-growing countries (Zhang et al., 2020; Kirdat et al., 2021). The symptoms of SCWL are similar to those of SCGS, namely leaf whitening, increased tillering, and dwarfing (Viswanathan et al., 2011; Kirdat et al., 2021). The phytoplasma isolates associated with SCWL and SCGS belong to the 16SrXI group based on the high similarity of their 16S rRNA gene sequences (Viswanathan et al., 2011; Abeysinghe et al., 2016). The group 16SrXI includes the subgroups 16SrXI-B, 16SrXI-D, and 16SrXI-F (Zhang et al., 2016; Yadav et al., 2017). In 2020, the draft SCGS phytoplasma genome was published (Kirdat et al., 2020), and based on its analysis, the phytoplasma isolates associated with SCGS have been classified as a novel taxon “*Candidatus* Phytoplasma sacchari” (Kirdat et al., 2021). Multilocus sequence typing revealed that SCWL and SCGS phytoplasmas belong to two different populations of “*Ca*. Phytoplasma sacchari” (Abeysinghe et al., 2016; Zhang et al., 2023).

With the development of sequencing technology, whole-genome sequencing of phytoplasmas has become feasible for many laboratories. Genome analysis is an efficient and effective approach to generate a significantly large amount of data for the biological characterization of unculturable bacteria. In this study, we developed a method for the enrichment of SCWL phytoplasma DNA for performing genome sequencing, and we obtained the complete genome sequence of the isolate by combining Illumina and Nanopore technologies. Our study will provide a simple method for the enrichment of phytoplasma genomic DNA and enhance our understanding of the genetic characteristics of the “*Ca*. Phytoplasma sacchari” species, thus providing a basis for research on its pathogenic mechanisms and other aspects.

## Materials and methods

### Source of phytoplasma

Sugarcane (*Saccharum officinarum* L.) samples exhibiting SCWL symptoms were collected from Lincang, Yunnan province, China, in 2018. They were maintained and propagated in an insect-proof greenhouse at the Sugarcane Research Institute, Yunnan Academy of Agricultural Sciences. We used the ROC22 sugarcane variety in this study.

### Extraction of genomic DNA from leaves

Genomic DNA from sugarcane leaves was extracted using the SDS method (Lim et al., 2016). The extracted DNA was detected using 1% agarose gel electrophoresis and quantified using a Qubit^®^ 3.0 Fluorometer (Invitrogen, USA).

### Enrichment of SCWL phytoplasma DNA

Approximately 5 g of sugarcane leaves were cut into small pieces using scissors and ground to obtain homogenate in 1 × PBS buffer (Sangon Biotech Co., Ltd., Shanghai, China). The homogenates were placed in 50 ml centrifuge tubes and centrifuged at 12,000 rpm for 5 min. The supernatant was discarded, and the pellet was resuspended in 50 ml of 1 × PBS buffer; this step was repeated thrice. The suspension was sequentially filtered through 100, 70, 40, 10, and 5 μm filters (Erwu Industrial Co., Ltd., Shanghai, China). The filtrate was centrifuged at 12,000 rpm for 5 min, and the supernatant was discarded; 20 μl of DNase I (3 units/μl) (TransGen Biotech Co., LTD, Beijing, China), 20 μl of 10 × DNase I Reaction Buffer, and 200 μl of ddH_2_O were added to the pellet and mixed well. Next, the pellet was incubated at 37°C for 10 min, and then 40 μl of EDTA (25 mmol/L) was added and incubated at 65°C for 10 min. The obtained solution was centrifuged at 12,000 rpm for 5 min, the supernatant was discarded, and the pellet was used to extract DNA using an Ezup Column Bacteria Genomic DNA Purification Kit (Sangon Biotech Co., Ltd., Shanghai, China), according to the manufacturer's instructions. Three biological replicates were performed.

### Library preparation and sequencing

Both Illumina short-read and Nanopore long-read sequencing technologies were used for genome sequencing. For Illumina, 0.2 μg of enriched DNA was used as the input material for DNA library preparations. The sequencing library was generated using a NEBNext^®^ Ultra™ DNA Library Prep Kit for Illumina (NEB, USA), according to the manufacturer's instructions. The DNA libraries were sequenced on an Illumina NovaSeq 6000 platform (Illumina, San Diego, USA), and 150 bp paired-end reads were generated. For Oxford Nanopore Technology (ONT) sequencing, 2.5 μg of total DNA was used as the input material for the DNA library preparations. The sequencing library was prepared using an ONT Ligation Kit (SQK-LSK109), followed by PromethION sequencing (ONT, Oxford, UK).

### Genome assembly and annotation

The Unicycler v 0.5.0 software (Wick et al., 2017) was used to assemble the filtered reads. First, highly accurate Illumina data (Q30 > 85%) were used for assembly to obtain high-quality genome contigs. Second, the Nanopore data were used to connect the high-quality contigs with a complete genome. Finally, the Pilon software (Walker et al., 2014) was used to correct the assembled genome using the Illumina data to obtain the final genome sequence with higher accuracy. The Illumina sequences were mapped to the SCWL1 genome using BWA v.0.7.17 (Li and Durbin, 2009) to evaluate the effect of SCWL phytoplasma DNA enrichment. Bamdst was used to analyze the depth of sequencing. Genome annotation was performed using Prokka v1.14.6 (Seemann, 2014), which comprises Prodigal, Aragorn, RNAmmer, and Infernal that predict open reading frames (ORFs), tRNAs, rRNAs, and ncRNA, respectively. KEGG (Kyoto Encyclopedia of Genes and Genomes), COG (Cluster of Orthologous Groups of proteins), NR (Non-Redundant Protein), UniProt (Unified Protein), GO (Gene Ontology), Pfam (Protein families), RefSeq (Reference Sequence), and TIGRFAMs databases were used for functional annotation of the genome.

### Phylogenetic analysis

For phylogenetic analysis, 14 complete phytoplasma genomes and the draft genome of “*Ca*. Phytoplasma sacchari” isolate SCGS (Supplementary Table 1) were compared. The homologous gene clusters were identified using OrthoMCL (Li et al., 2003). Multiple sequence alignments of single-copy homologous gene clusters were prepared using MUSCLE (Edgar, 2004) and concatenated to produce one super alignment matrix. The resulting multiple sequence alignment was used to build a phylogenetic tree using the maximum likelihood method implemented in MEGA X (Kumar et al., 2018). The average nucleotide identity (ANI) was calculated using the orthoANI tool of EzBioCloud (https://www.ezbiocloud.net/tools/ani) (Yoon et al., 2017). The digital DNA–DNA hybridization values were calculated using the Genome-to-Genome Distance Calculator (GGDC 3.0; https://ggdc.dsmz.de/ggdc.php#) (Meier-Kolthoff et al., 2022).

## Results

### General features of the genome of “*Ca*. phytoplasma sacchari” isolate SCWL1

**Figure 1:**
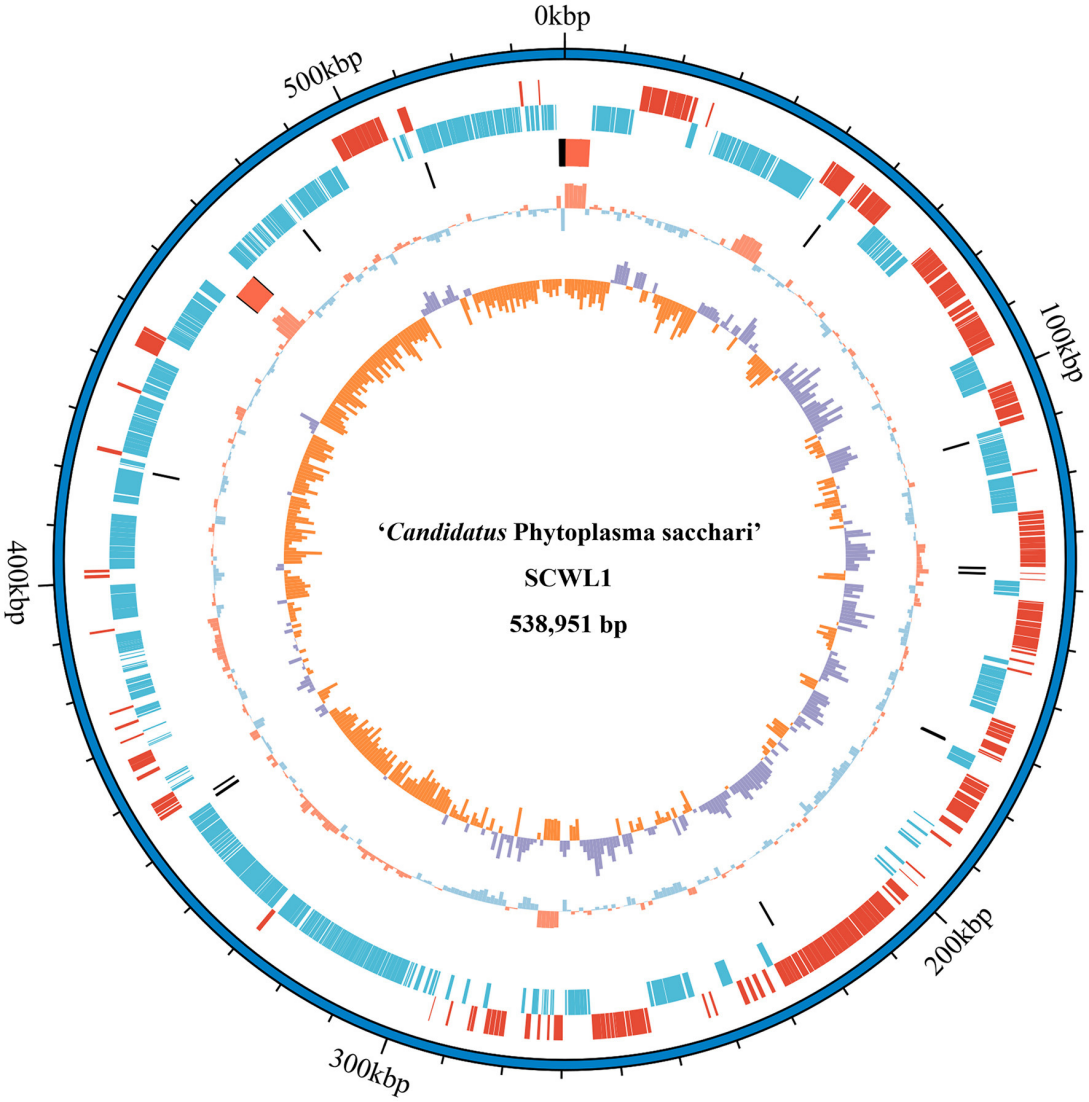
Circular representation of the chromosome of “*Candidatus* Phytoplasma sacchari” isolate SCWL1. From outer to inner rings: (1) Scale marks. (2 and 3) Coding sequences on the forward and reverse strands, respectively. (4) rRNA genes (red) and tRNA genes (black). (5) GC content (above-average: red; below-average: blue). (6) GC skew index (positive: purple; negative: orange).

Our analysis revealed that the genome of “*Ca*. Phytoplasma sacchari” isolate SCWL1 was composed of a circular chromosome and a plasmid comprising 538,951 bp with 20.54% G+C content (Figure 1) and 2,976 bp with 21.00% G+C content, respectively. The chromosome contained 459 coding sequences (CDSs), two complete 5S-23S-16S rRNA gene operons, and 27 tRNA genes (Table 1 and Figure 1). The sequence identity between the two 16S rRNA gene sequences was 100%. The total length of the CDS was 413,403 bp, and the average length was 901 bp, accounting for 76.71% of the total length of the chromosome.

**Table 1:** General features of the genome of “*Candidatus* Phytoplasma sacchari” isolate SCWL1.

**Gene type**	**Number**	**Total length**	**Average length**	**Percentage of genome (%)**
Gene	500	425,685	851	78.98
CDS	459	413,403	901	76.71
tRNA	27	2,152	80	0.40
23S rRNA	2	5,723	2,862	1.06
16S rRNA	2	3,046	1,523	0.57
5S rRNA	2	232	116	0.04
misc RNA	8	1,129	141	0.21

misc RNA, miscellaneous RNA.

### Evaluation of isolate SCWL DNA enrichment method

**Figure 2:**
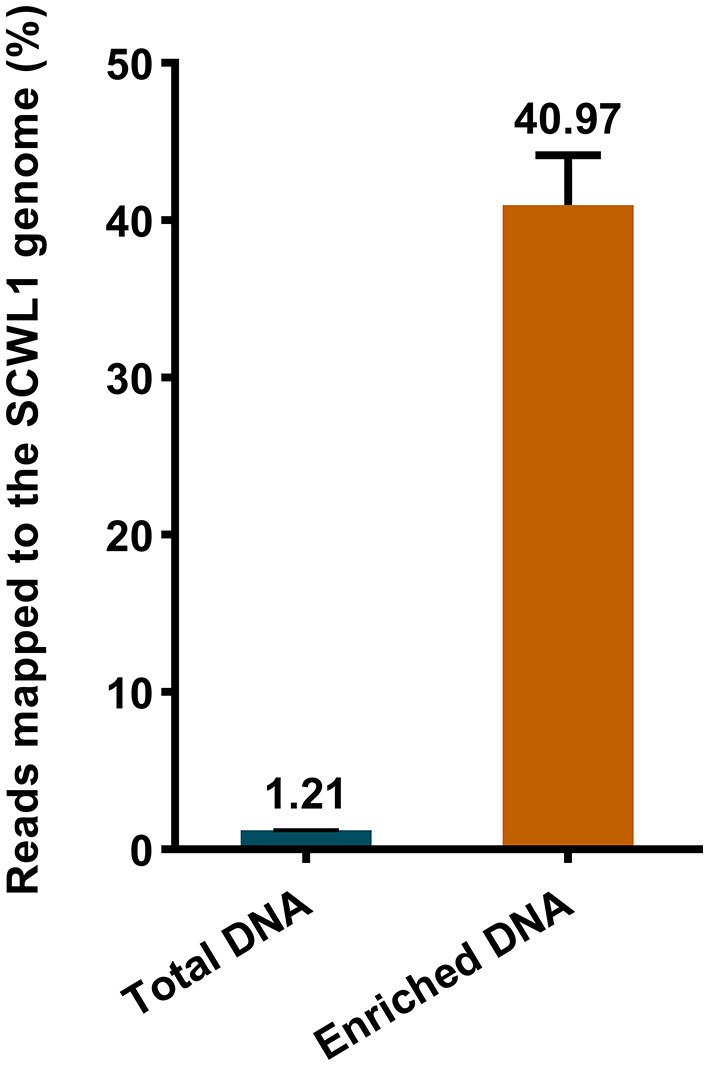
Percentage of sequence reads mapped to the genome of “*Candidatus* Phytoplasma sacchari” isolate SCWL1.

We evaluated the efficacy of the SCWL phytoplasma DNA enrichment method for Illumina sequencing. The enriched DNA and total DNA from leaves were sequenced using Illumina sequencing. After quality-control assessment of enriched DNA and total DNA sequencing reads, an average of 4,219,460 and 16,869,320 clean reads were obtained, respectively (Table 2). Only an average of 204,417 reads from the total DNA were mapped to the SCWL1 genome, accounting for only 1.21% of all clean reads, whereas an average of 1,744,476 reads from the enriched DNA were mapped to the SCWL1 genome, accounting for 40.97% of all clean reads (Figure 2). The highest sequence coverage from total DNA was 99.13% and that from enriched DNA was 100% (Table 2).

**Table 2:** Illumina sequencing data.

**Run^a^**	**Raw reads**	**Raw base (G)**	**Clean reads**	**Clean base (G)**	**Effective rate (%)**	**Sequencing depth (fold)**	**Reads mapped to SCWL1 genome**	**Coverage (%)**
C1	18,032,029	5.41	17,930,551	5.38	99.44	61.48	220,891	99.13
C2	15,799,153	4.74	15,687,968	4.71	99.30	52.04	186,987	99.06
C3	17,098,873	5.13	16,989,440	5.08	99.36	57.16	205,372	99.11
S1	6,060,382	1.82	5,235,443	1.57	86.39	733.85	2,322,713	100
S2	3,929,251	1.18	3,528,420	1.06	89.80	364.14	1,429,359	100
S3	4,428,736	1.33	3,894,516	1.17	87.94	358.57	1,481,357	100

^a^Runs C1–C3: Illumina sequencing of total leaf DNA, with three biological replicates; Runs S1–S3: Illumina sequencing of enriched DNA, with three biological replicates.

### Functional annotation for the protein-coding genes

**Figure 3:**
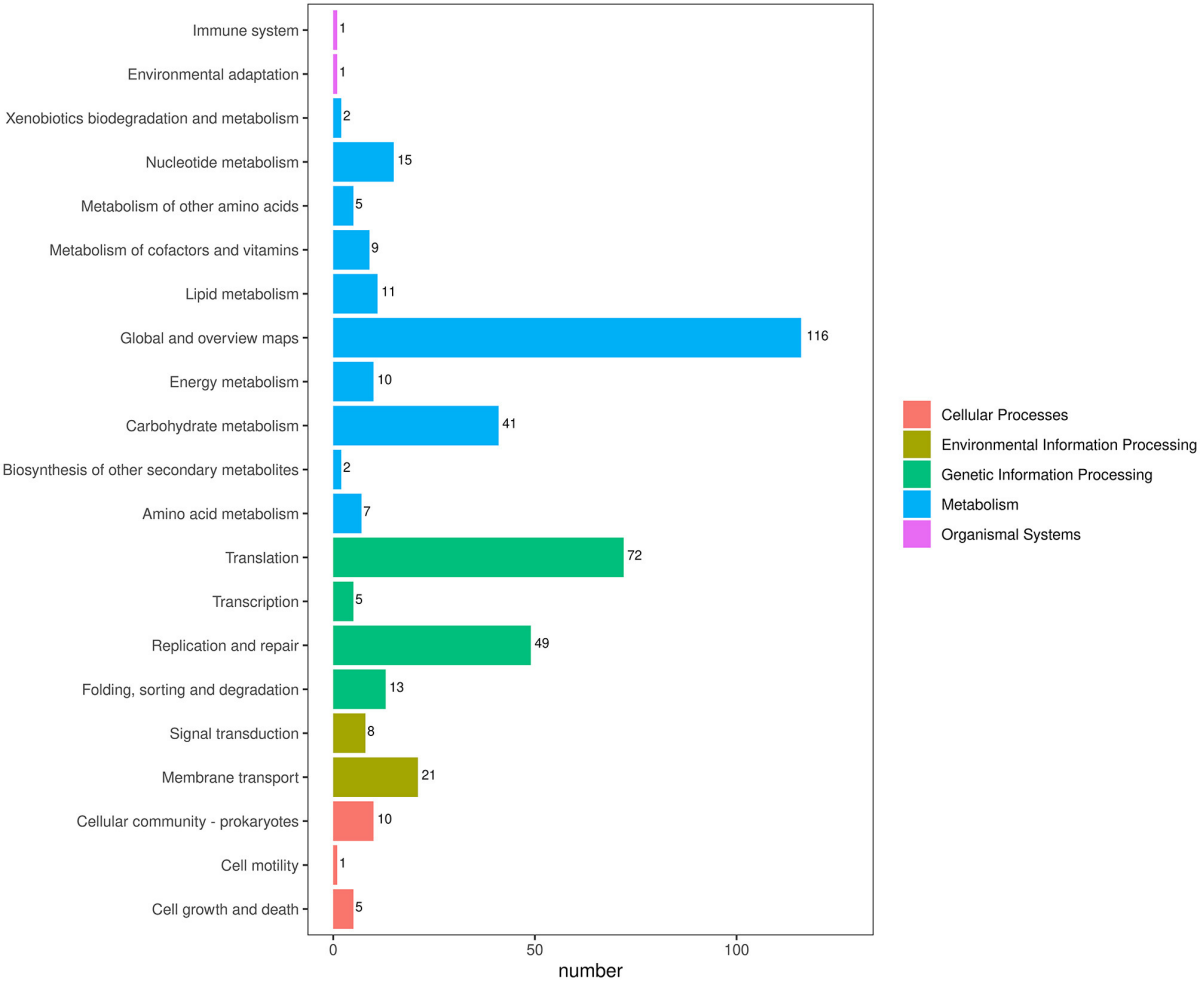
Classification map of Kyoto Encyclopedia of Genes and Genomes (KEGG) pathway annotation analysis of the genome of “*Candidatus* Phytoplasma sacchari” isolate SCWL1.

**Figure 4:**
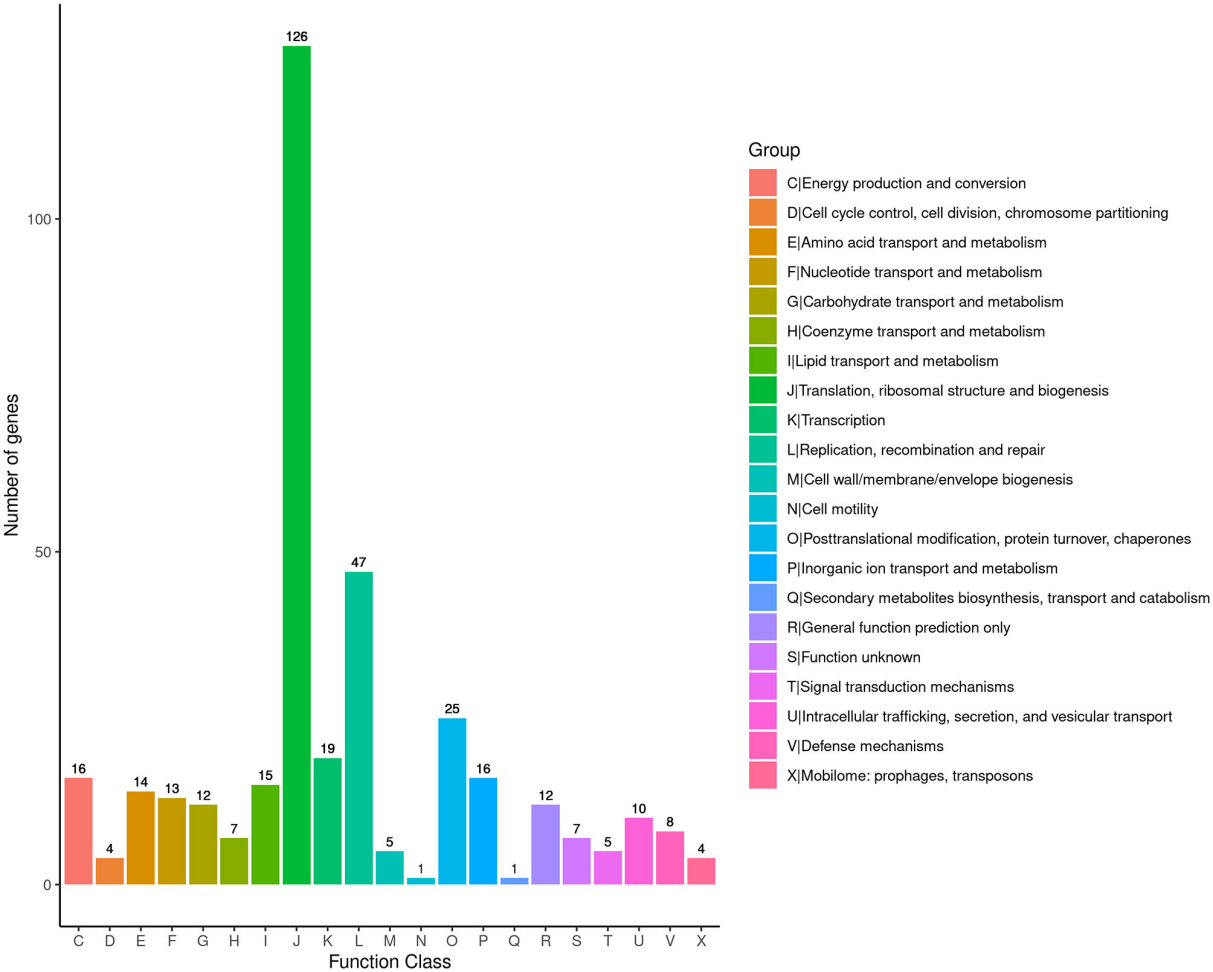
Classification map of Cluster of Orthologous Groups of proteins (COG) annotation analysis of the genome of “*Candidatus* Phytoplasma sacchari” isolate SCWL1.

**Figure 5:**
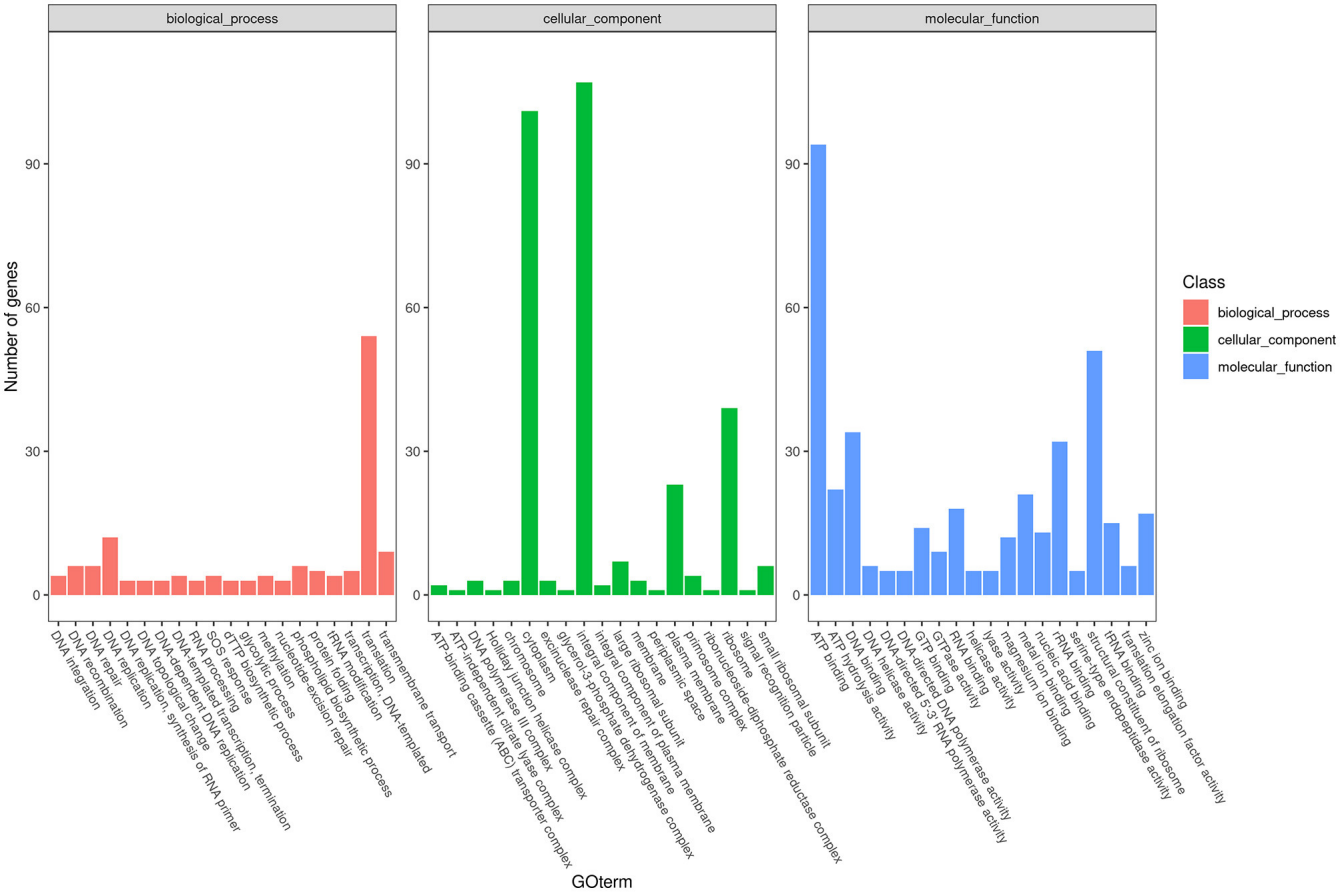
Classification map of Gene Ontology (GO) annotation analysis of the genome of “*Candidatus* Phytoplasma sacchari” isolate SCWL1.

To obtain comprehensive information on gene function, the protein-coding genes in the SCWL1 genome were annotated using eight databases (Supplementary Table 2). Two hundred genes were annotated using the KEGG database and classified according to the KEGG pathway (Figure 3). The maximum number of genes in metabolism were enriched in global and overview maps (116 genes) and carbohydrate metabolism (41 genes); in genetic information processing, they were enriched in translation (72 genes) and replication and repair (49 genes). Three hundred and forty-eight genes were annotated using the COG database and assigned to 21 functional categories (Figure 4). The most abundant functional class was COG class J (translation, ribosomal structure, and biogenesis). Based on the GO database, we annotated 378 genes, which were categorized into three functional categories (biological process, cellular component, and molecular function). The top 20 GO terms with the most annotations of each functional category are shown in Figure 5. The most enriched biological process, cellular component, and molecular function terms were translation, integral component of plasma membrane and cytoplasm, and ATP binding, respectively. The highest number of genes was annotated in the Nr database (424 genes) and the RefSeq database (424 genes). In the NR database, 389 genes were annotated to the genome of “*Ca*. Phytoplasma sacchari,” accounting for 91.75% of all annotated genes.

### Metabolic pathways

**Figure 6:**
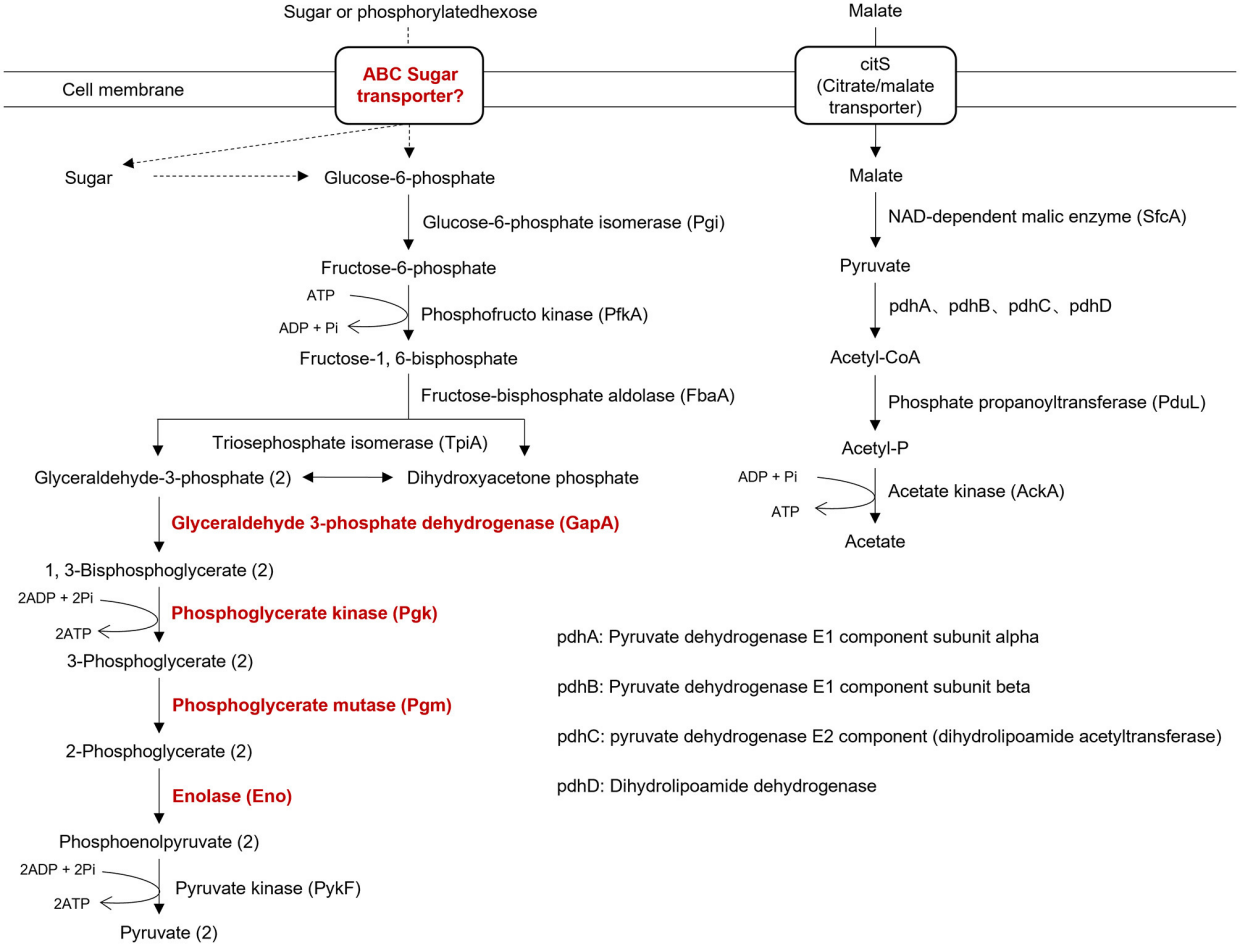
Annotation of genes related to energy-yielding pathways in the genome of isolate SCWL1. The proteins marked in red indicate that they are not absent in the genome of isolate SCWL1 genome; the dotted line represents the absence of the enzyme involved in the reaction.

Like other phytoplasma isolates, isolate SCWL1 lacked many genes encoding the tricarboxylic acid cycle, epoxidative phosphorylation, pentose phosphate pathway, and F1F0 ATP synthase. The genes encoding the phosphoenol pyruvate-dependent sugar phosphotransferase system (PTS), hexokinase, and sugar transport system (*malE, malG*, and *malF*) were also absent. Similar to that in the genome of “*Ca*. Phytoplasma mali” isolate AT, only five glycolysis-related genes (*Pgi, PfkA, FbaA, TpiA*, and *PykF*) were present in the genome of isolate SCWL1 (Figure 6). Although the SCWL1 genome did not possess glycolytic pathway-related genes, the genes encoding malate or citrate transporter protein (*citS*), malic enzyme (*sfcA*), pyruvate dehydrogenase multienzyme complex (*pdhA, pdhB, pdhC*, and *pdhD*), and a putative phosphate propanoyl transferase (*pduL*) were present (Figure 6). In addition, the citrate lyase gene clusters *(citXFEDG)* encoding the apo-citrate lyase phosphoribosyl-dephospho-CoA transferase *(citX)*, the α-subunit *(citF)*, the β-subunit *(citE)* and the γ-subunit *(citD)* of citrate lyase, and 2-(5′-triphosphoribosyl)-3′-dephosphocoenzyme-A synthase (*citG*) is found in SCWL1 genomes.

### Potential mobile units (PMUs) and effector genes

**Figure 7:**
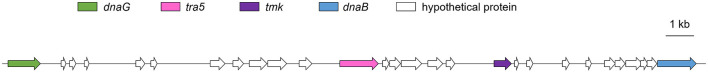
Potential mobile units (PMUs) in the genome of “*Candidatus* Phytoplasma sacchari” isolate SCWL1. dnaG, DNA primase; tra5, IS3 family transposase; hflB, ATP-dependent Zn protease; tmk, thymidylate kinase; dnaB, replicative DNA helicase.

PMUs are commonly found in phytoplasma genomes. A PMU, with a size of 23.6 kb, consisting of *tra5, tmk, dnaB*, and *dnaG* was found in the genome of isolate SCWL1 (Figure 7 and Supplementary Table 3). Other core genes of the phytoplasma PMU region, such as *ssb, rpoD*, and *himA*, were scattered throughout the genome of the isolate. In the PMU region, two incomplete *hflB* genes and one incomplete *dnaG* gene were annotated. Proteins homologous to phytoplasma effectors, such as TENGU, SAP05, SAP11, and SAP54, were not found in the genome of isolate SCWL1.

### Phylogenetic relationships

**Figure 8:**
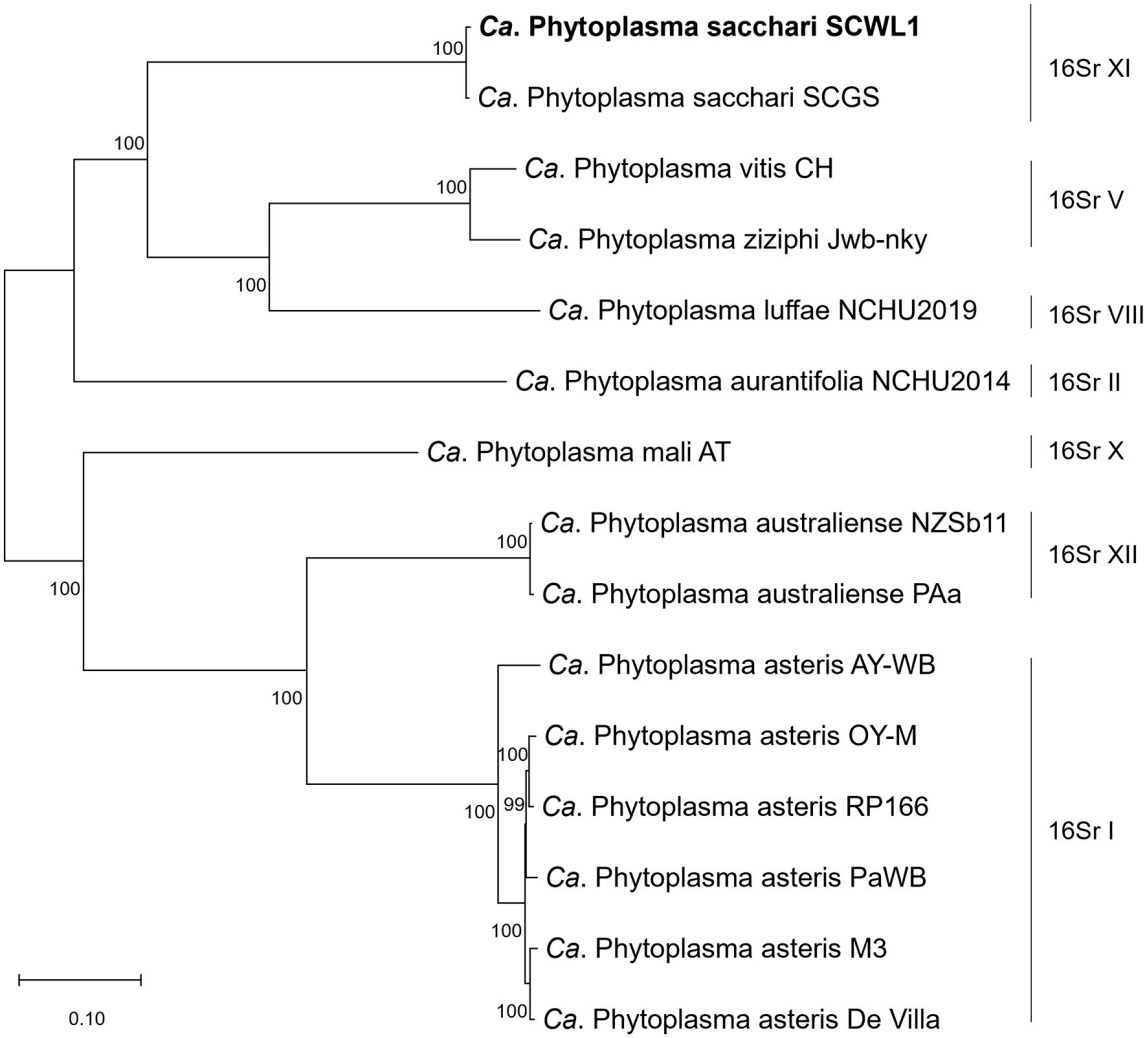
Maximum likelihood phylogeny of phytoplasmas inferred based on a concatenated alignment of 191 single-copy orthologous proteins.

The comparative analysis of isolate SCWL1 and 14 phytoplasma genomes revealed the presence of 191 single-copy orthologous proteins. The phylogenetic tree constructed based on the concatenated sequences of these single-copy proteins elucidated that isolate SCWL1 was most closely related to isolate SCGS (Figure 8). Comparison analysis of 16S rRNA gene sequences (full length) indicated that isolates SCWL1 and SCGS shared 99.87% sequence identity. At the whole-genome level, the ANI value for isolate SCGS against isolate SCWL1 was 98.80%, and the digital DNA–DNA hybridization (dDDH) value was 89.50%.

## Discussion

In the present study, we developed a novel method for enriching the DNA of phytoplasma isolate SCWL, which is simpler and faster than the previously established methods. In brief, the method is as follows: First, the SCWL phytoplasma was released by grinding the sugarcane leaves. Second, the host DNA released during the grinding process was removed by washing several times; the host tissues and cells were removed *via* serial filtration; and the residual host DNA was digested using DNase I. Finally, the genomic DNA from the filtered SCWL phytoplasma cells was extracted. The results of Illumina sequencing revealed that the number of SCWL phytoplasma reads for enriched DNA was significantly increased, and data such as average sequencing depth and coverage were better than those obtained using total leaf DNA. Although up to 40.97% of the reads obtained *via* the sequencing of enriched DNA were mapped to the genome of isolate SCWL1, non-SCWL phytoplasma reads still accounted for a large fraction of the total reads. The reason for this result could be that many endophytic microorganisms and host plant organelles were present during the final DNA extraction step, i.e., the filtration process. Although this is a limitation of the enrichment method developed in this study, the method does not require expensive equipment and reagents and is convenient and fast, and the enriched DNA can meet Illumina sequencing requirements.

Recently, with the reduction in sequencing costs and the development of numerous sequencing technologies, such as NGS, phytoplasma genomes can be easily sequenced and larger sequencing data can be generated with limited funds, thereby achieving higher coverage and generating a more complete genome draft. The emergence of third-generation sequencing technology has enabled the generation of longer read lengths, making genome assembly easier. Phytoplasma genomes are rich in repeated DNA sequences, thus making genome assembly difficult using only second-generation sequencing data. In this study, although the coverage of Illumina sequencing using enriched DNA was 100%, the assembly of the genome of isolate SCWL1 was unsuccessful using only Illumina sequencing data. Recently, several complete phytoplasma genomes have been generated by combining second- and third-generation sequencing technologies (Wang et al., 2018; Debonneville et al., 2022; Huang et al., 2022). In this study, although enriched DNA was used for second-generation sequencing, it was not suitable for third-generation library preparation and sequencing due to low DNA concentration; therefore, we performed Nanopore sequencing using total leaf DNA. In this study, although the combination of second- and third-generation sequencing of phytoplasma genomes does not require genome enrichment, Illumina sequencing reads using the total leaf DNA did not completely cover the genome of isolate SCWL1. The accuracy of Nanopore sequencing is lower than that of Illumina sequencing, suggesting that the accuracy of the assembled genome assembly will be reduced if only Nanopore sequencing data are used. Therefore, appropriate enrichment of phytoplasma DNA is essential for phytoplasma genome sequencing.

Initially, the size of phytoplasma genomes was estimated to be 530–1350 kb with 21–33% GC content (Neimark and Kirkpatrick, 1993; Marcone et al., 1999; IRPCM Phytoplasma/Spiroplasma Working Team-Phytoplasma Taxonomy Group, 2004). Recent studies have reported the size of complete phytoplasma genomes to be 576–960 kb (Wei and Zhao, 2022). In this study, the size of the SCWL1 chromosome is 538,951 bp, which is the smallest complete phytoplasma chromosome reported, and the predicted GC content and the number of coding genes are also the least among all complete phytoplasma genomes that have been reported. Similar to that of the “*Ca*. Phytoplasma mali” isolate AT, the chromosome of isolate SCWL1 exhibited a regular cumulative GC-skew pattern. Due to the lack of all glycolysis-related genes in the genome of “*Ca*. Phytoplasma mali” isolate AT, it is proposed that malate is utilized as carbon and energy sources (Kube et al., 2008). In this study, the genome of isolate SCWL1 also lacked the glycolysis-related enzymes, but the enzymes encoding the conversion pathway for malate conversion to acetate were present. Therefore, it is possible that isolate SCWL1 does not rely on glycolysis for energy production, and the malate-to-acetate pathway is an alternative to glycolysis and the main pathway for isolate SCWL1 to obtain carbon sources and produce energy.

With advancements in genome sequencing technology, the classification and phylogeny of phytoplasmas based on whole-genome sequence can be elucidated. The accepted minimum threshold for taxon assignment in prokaryotes using genomic data is that the same species isolates should have ANI values >95–96% and DDH values >70% (Richter and Rosselló-Móra, 2009; Chun et al., 2018). In 2022, the revised version of the guidelines for defining “*Ca*. phytoplasma” species proposed a whole-genome ANI standard of 95% for “*Ca*. phytoplasma” species delineation (Bertaccini et al., 2022). Previous studies have proposed isolate SCGS as a novel taxon “*Ca*. Phytoplasma sacchari” (Kirdat et al., 2021). Recently, multilocus sequence typing revealed that SCGS and SCWL phytoplasma isolates belonged to different populations of “*Ca*. Phytoplasma sacchari,” but the classification was made without any genomic-level evidence (Abeysinghe et al., 2016; Zhang et al., 2023). In this study, we analyzed the phylogenetic relationships between isolates SCGS and SCWL at the genomic level and found that the ANI and dDDH values between their genomes were higher than the threshold values for taxon assignment of “*Ca*. phytoplasma” species. Since the genomes of only two “*Ca*. Phytoplasma sacchari” isolates are available, for further elucidation of the evolutionary relationship and population structure of “*Ca*. Phytoplasma sacchari,” genome sequencing of more isolates belonging to this genus is required; this can be performed using the enrichment method developed in this study.

## Conclusion

To improve the efficiency of phytoplasma sequencing, a filter-based enrichment method for the genome of phytoplasma isolate SCWL was developed. The method increased the number of phytoplasma sequences obtained *via* Illumina sequencing. This method will not only help in the initiation of more “*Ca*. Phytoplasma sacchari” genome sequencing projects but also act as an important reference for the enrichment of the genome DNA of other phytoplasma species. The genome sequence of isolate SCWL1 is the first complete genome sequence of a phytoplasma isolate belonging to the 16SrXI group, thus promoting an in-depth understanding of the genomic characteristics of the 16SrXI group. Moreover, the chromosome of “*Ca*. Phytoplasma sacchari” isolate SCWL1 is the smallest circular chromosome among the phytoplasma with complete genome sequences available. This study also provides genomic evidence that isolates SCGS phytoplasma and SCWL belong to the same phytoplasma species. The availability of the complete genome of isolate SCWL1 will contribute to future studies on the molecular evolution and pathogenesis of “*Ca*. Phytoplasma sacchari.”

## Data availability statement

The datasets presented in this study can be found in online repositories. The names of the repository/repositories and accession number(s) can be found in the article/Supplementary material.

## Author contributions

R-YZ and X-HH conceived and designed the experiment, analyzed the data, and wrote the manuscript. X-YW, JL, H-LS, and Y-HL performed the experiment and participated in the data analysis. Y-KH acquired the funding, supervised the project, and revised the manuscript. All authors contributed to the article and approved the submitted version.
